# News media narratives of Covid-19 across 20 countries: Early global convergence and later regional divergence

**DOI:** 10.1371/journal.pone.0256358

**Published:** 2021-09-01

**Authors:** Reuben Ng, Ting Yu Joanne Chow, Wenshu Yang

**Affiliations:** 1 Lee Kuan Yew School of Public Policy, National University of Singapore, Singapore, Singapore; 2 Lloyd’s Register Institute for the Public Understanding of Risk, National University of Singapore, Singapore, Singapore; The University of Hong Kong, HONG KONG

## Abstract

**Background:**

Seldom in history does one get a ‘front row seat’—with large-scale dynamic data—on how online news media narratives shift with a global pandemic. News media narratives matter because they shape societal perceptions and influence the core tent poles of our society, from the economy to elections. Given its importance—and with the benefit of hindsight—we provide a systematic framework to analyze news narratives of Covid-19, laying the groundwork to evaluate policy and risk communications.

**Objectives:**

We leverage a 10-billion-word-database of online news, taken from over 7,000 English newspapers and magazines across 20 countries, culminating in 28 million articles. First, we track the volume of Covid-19 conversations across 20 countries from before to during the pandemic (Oct’19 to May’20). Second, we distill the phases of global pandemic narratives, and elucidate regional differences.

**Methods:**

To track the volume of Covid-19 narratives, we identified 10 target terms—Coronavirus, Covid-19, Covid, nCoV, SARS-CoV-2, Wuhan Virus, Virus, Disease, Epidemic, Pandemic—and tracked their combined monthly prevalence across eight months from October 2019 through May 2020. Globally, across 20 countries, we identified 18,042,855 descriptors of the target terms. Further, these descriptors were analysed with natural language processing models to generate the top five topics of Covid-19 that were labelled by two independent researchers. This process was repeated across six continents to distil regional topics.

**Results:**

Our model found four phases of online news media narratives: Pre-pandemic, Early, Peak and Recovery. Pre-pandemic narratives (Oct’19–Dec’19) were divergent across regions with Africa focused on monkeypox, Asia on dengue fever, and North America on Lyme disease and AIDS. Early (Jan–Feb’20) and Peak Pandemic (Mar–May’20) evidenced a global convergence, reflecting the omnipresence of Covid-19. The brief transition from early to peak pandemic narratives underscored the pandemic’s rapid spread. Emerging from the embers of the pandemic’s peak were nascent recovery words that are regionally divergent—Oceania focused on hope and an uncertain future while North America centered on re-opening the economy and tackling discrimination.

**Conclusions:**

Practically, we presented a media barometer of Covid-19, and provided a framework to analyse the pandemic’s impact on societal perceptions—laying the important groundwork for policy makers to evaluate policy communications, and design risk communication strategies.

## Introduction

While there has been an exponential increase in publications devoted to the pandemic, albeit mostly epidemiologic and medical, few have taken a systematic approach to analyze online news media narratives of Covid-19 across 20 countries. Such studies are valuable for policy makers as they lay the groundwork to evaluate policy communications and design risk communication strategies. We leveraged a 10-billion-word database of online English news media, taken from over 7000 newspapers and magazines, culminating in 28 million articles from 20 countries—to study the phases of Covid-19 narratives globally, and regionally.

Our study contributes to the Covid-19 social sciences literature in the following ways. First, we dynamically track online news media narratives, from before to during the pandemic—month-by-month from 1^st^ October 2019 to 31^st^ May 2020—across 20 countries. Most Covid-19 perception studies are survey-based, and cross-sectional—measuring a static snapshot of societal perceptions. Given the fast-moving nature of the pandemic globally, and the flurry of evolving government interventions locally, our month-by-month analysis over 8 months provides a systematic picture of how news media narratives have shifted in the pandemic.

Second, our study is expansive in scale and scope. Existing early Covid-19 perception studies fall into four general categories: Focused on specific groups such as nursing staff [1], patients with existing medical conditions [2], medical students [3], and dentists [4]; Limited in geographic focus with convenient sampling of survey participants within single countries such as the US, UK, Iran, Egypt [5–7]; Focused on specific topics, such as perceptions towards risk [8], online learning [9], or ageism [10–12]; Representative surveys on various topics from specific countries or regions, employment and mental health in the United States [13]. Building on these important foundations, our study is global in nature—covering 20 countries—and does not a priori restrict to certain groups or topic, using a comprehensive 10-billion-word platform to delineate online news media narratives. This is paramount as Covid-19 has exerted a wide and deep impact on society, and our study seeks to distill its impact on narratives.

Third, and from a practical standpoint, news media are important as they shape societal perceptions. Given the importance of how events are portrayed in the media—and with the benefit of hindsight—we provide a coherent overview of news media narratives during a fast-moving and wide-reaching pandemic. Such a framework is important to both scholars and policymakers as it lays the groundwork to evaluate policy communications and design better risk communication strategies. The significance of this work is underscored by theories like *agenda setting* that highlight the media’s considerable influence on how issues gain public attention, and what specific issues have been deemed salient to a community of news readers by producers of such media [14]. Further, *mainstreaming*, under *cultivation theory*, asserts that producers of news media homogenize and create broad dimensions of shared meaning, particularly in a commercial environment within a capitalistic media system driven by clicks. As such, the process of mainstreaming certain topics magnifies its importance in the minds of an audience and overrides differences in perspectives for a homogenized narrative [15]. Together, these two processes contribute to a cyclical relationship [16], with cultivation overexposing and mainstreaming a certain topic, and agenda setting further pursuing it as an issue of importance—thus feeding back into its overexposure. The result of such media processes is reflected in how an audience perceives social issues. For instance, extended coverage on issues creates the impression that such issues are more important and credible [17], and such coverage may skew biases and perceptions [18], influence public opinion [19] and stock market shifts [20]. With the importance of media in shaping public perceptions and attitudes, we endeavor to investigate how virus narratives have unfolded throughout the early Covid-19 outbreak.

Specifically, our study seeks to achieve two goals. First, we track the global volume of Covid-19 conversations across 20 countries during the pandemic. Second, we distill the phases of global pandemic narratives and elucidate differences across six regions, and examine the temporal differences in narratives found pre-pandemic and during the pandemic.

## Materials and methods

### Dataset

We used the News on the Web corpus as our dataset: the largest cross-cultural English corpus collated from over 7000 online newspapers, magazines—culminating in 30 million articles across 20 countries [21]. The 20 countries span six regions: North America (America, Canada), Oceania (Australia, New Zealand), Asia (Bangladesh, Hong Kong, India, Malaysia, Pakistan, Philippines, Singapore, Sri Lanka), Africa (Ghana, Kenya, Nigeria, South Africa, Tanzania), Europe (Ireland, United Kingdom), and the Caribbean (Jamaica). The corpus is dynamic with 200 million words, from 300,000 new articles, added every month. Each country is represented by a wide variety of news sources; for instance, Australia is represented by hundreds of Australian news sites, with some examples of the most prominent local sources being *ABC Local/Online*, *Business Insider Australia*, *Gizmodo Australia*, *Huffington Post Australia*, *Kotaku Australia*, *Perth Now*, *The Sydney Morning Herald*, *The Australian Financial Review*, and *The Canberra Times*. Likewise, Jamaica is represented by local Jamaican news sites, with examples being *Jamaica Gleaner*, *Jamaica Observer*, *Loop News Jamaica*. These sites generally cover local and global news occurrences on a daily basis, with some specializing in business or technological news [22]. With an extensive range of news sources registered under each country’s domain code, the corpus serves to aggregate the general trends found in each country’s news media representation of virus narratives. This dataset was created with funding from the National Science Foundation (NSF) and the National Endowment for the Humanities (NEH) to study contemporary language usage in countries where English is widely used.

### Prevalence of Covid-19-related words across 20 countries

To track the volume of pandemic-related coverage, we identified 10 target words—*Coronavirus*, *Covid-19*, *Covid*, *nCoV*, *SARS-CoV-2*, *Wuhan Virus*, *Virus*, *Disease*, *Epidemic*, *Pandemic*—and tracked their combined monthly prevalence across eight months from October 2019 through May 2020. Similar to other studies [23], prevalence per month, by country, was calculated by the ratio of the number times all 10 target words appeared in the respective country’s dataset (numerator) and the total number words that were available in the respective country’s dataset (denominator). The ratio was multiplied by 1,000,000 to provide the prevalence rate of words per million. We tested the prevalence across eight months with an exponential model.

### Content of Covid-19 online news media coverage

To elucidate the content of Covid-19 narratives across 20 countries, our platform ingested the relevant online news media data across 20 countries from October 2019 through May 2020, culminating in 1.5 billion words. After pre-processing the corpus by excluding prepositions, conjunctions, and ‘stop’ words (e.g. *and*, *the*, *that*), we generated collocates (i.e. words that co-occurred most frequently with 10 target words) for each of the 20 countries, every month, between October 2019 to May 2020. These collocates had the following qualifying criteria: (a) Lexical Proximity: collocate present within six words prior or after the respective target word. Articles such as ‘the’, ‘a’ were not included in the six-word lexical span. If the target noun was the first word of a sentence, the collocates from the prior sentence were excluded; (b) Mutual Information Score of three and above: collocate had a stronger association with the respective synonym compared to other words in the corpus for that country, indicating semantic bonding [24]. This is an application of computational linguistics to study topic content, and language shifts in other studies [10, 23, 42–44]. The rigorous process culminated in 18,042,855 collocates selected for analysis over all eight months, across 20 countries.

Thereafter, we conducted Latent Dirichlet Allocation (LDA), an unsupervised natural language processing algorithm to find the most salient topics, by grouping collocates that most probabilistically appear together in the same context. LDA is robust and valid, given the statistical comparability to manual topic labelling [25] and its appropriateness as a tool to analyze granular topics related to an event over time [26]. Globally, across 20 countries, the analysis identified the top five topics per month, that were labelled by two independent researchers where inter-rater reliability using Cronbach’s alpha was 0.87 (95% CI: 0.82, 0.92), from October 2019 through May 2020. Regionally, we distilled the top five topics per month for each of the six regions, also labelled by two independent researchers.

## Results

### Global volume of Covid-19 narratives across 20 countries

**Fig 1:**
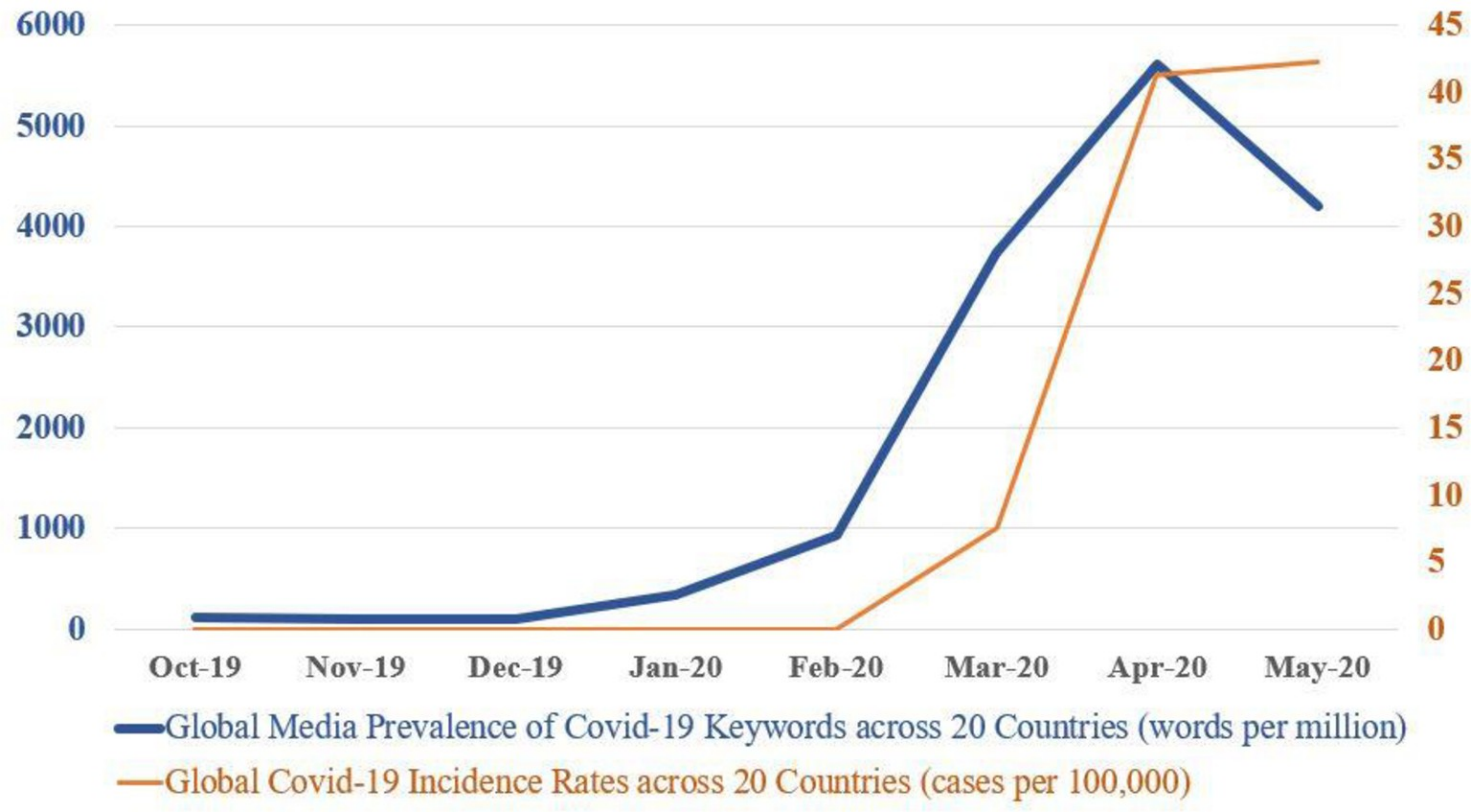
Volume of global Covid-19 narratives & Covid-19 incidence rates. Prevalence of 10 virus-related keywords (Coronavirus, Covid-19, Covid, nCoV, SARS-CoV-2, Wuhan Virus, Virus, Disease, Epidemic, Pandemic) experienced exponential growth from a baseline of 100 words per million in October 2019 to 5500 words per million in April 2020—a 55-fold increase. The prevalence of global Covid-19 narratives dovetails the global Covid-19 incidence rate across 20 countries.

The prevalence of the 10 virus-related keywords (Coronavirus, Covid-19, Covid, nCoV, SARS-CoV-2, Wuhan Virus, Virus, Disease, Epidemic, Pandemic*)* from October-December 2019 averaged a baseline of 100 words per million, and increased to 5,500 words per million in April 2020—a 55-fold increase—before dipping slightly to over 4,000 words per million in May (Fig 1). The increase in prevalence from Oct’19-Apr’20 followed a statistically significant exponential growth, given by the growth rate constant R_0_ (0.709±0.113, *P* = .009).

### Four phases of global Covid-19 narratives

**Fig 2:**
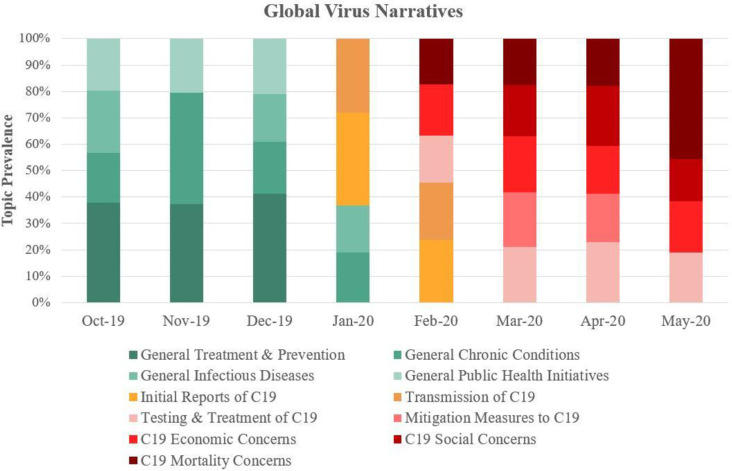
Global Covid-19 narratives across 20 countries. Pre-pandemic narratives (Oct’19–Dec’19; coded ‘green’) focused on general infectious disease topics of prevention and treatment. Early pandemic narratives (Jan’20–Feb’20; coded ‘orange’) were breaking news of Covid-19. The brief transition from Early to Peak Pandemic narratives (Mar’20–May’20; coded ‘red’) underscored the pandemic’s rapid spread across six regions. Peak pandemic narratives included testing, societal risk and vulnerability, mitigation and lockdown, economic crisis, death.

The global analysis of Covid-19 topics across 20 countries distilled four phases (Fig 2): *Pre-Pandemic* (Oct–Dec 2019), *Early Pandemic* (Jan–Feb 2020), *Peak-Pandemic* (Mar–May 2020).

Specifically, Phase 1, the *pre-pandemic* phase (Table 1), contained four subtopics about general disease-related topics that were evenly distributed: Treatment and Prevention (38%), Infectious Diseases (23%), Public Health Initiatives (20%), and Chronic Conditions (19%). The pre-pandemic phase provided a baseline for distribution of disease-related topics in news media, and the target words’ combined prevalence was 107 per million.

**Table 1:** Pre-pandemic topics and collocates.

Treatment and Prevention	*drug*, *treatment*, *medical*, *immunization*, *cure*, *treat*, *diagnose*, *prevent*, *patient*, *therapy*, *vaccine*
Infectious Diseases	*outbreak*, *infectious*, *virus*, *bacteria*, *infect*, *transmit*, *risk*, *contagious*, *spread*, *control*
Public Health Initiatives	*public*, *awareness*, *preventable*, *legislation*, *concern*, *tackle*, *ministry*, *response*, *raise*, *coalition*
Chronic Conditions	*diabetes*, *stroke*, *cancer*, *dementia*, *disease*, *chronic*, *cardiovascular*, *coronary*, *pulmonary*, *heart*, *lung*, *liver*, *kidney*

Phase 2 (Jan-Feb 2020): The *Early Pandemic* phase (Table 2) captured the ‘breaking news’ nature of what would rapidly become a global pandemic. It consisted of two subtopics: Emerging Reports of Covid-19 (35%) and Transmission Characteristics (28%). Between January and February 2020, these subtopics quickly dominated global news narratives, making up 63% of health-related topics. The target words’ combined prevalence grew to 644 per million.

**Table 2:** Early pandemic topics and collocates.

Emerging Reports of Covid-19	*coronavirus*, *Chinese*, *province*, *report*, *outbreak*, *alert*, *confirm*, *novel*, *director*, *epicentre*, *originate*, *emerge*, *center*, *bat*
Transmission Characteristics	*coronavirus*, *spread*, *infectious*, *contact*, *cause*, *symptom*, *transmission*, *transmit*, *infect*, *measure*, *detect*

Phase 3 (March–May 2020): the *Peak Pandemic* phase (Table 3) converged solely on Covid 19, with five subtopics that included Testing (23%), Societal Risk and Vulnerability (23%), Mitigation and Lockdown (18%), Economic Crisis (18%), Death (18%). The Covid-19 target words’ combined prevalence was peaked at 4518 per million, a seven-fold increase from the early pandemic phase.

**Table 3:** Peak pandemic topics and collocates.

Testing of Covid-19	*coronavirus*, *vaccine*, *hospital*, *patient*, *test*, *testing*, *diagnose*, *positive*, *negative*, *medical*, *care*, *dr*, *symptom*
Societal Risk and Vulnerability	*frontline*, *staff*, *healthcare*, *health*, *worker*, *risk*, *care*, *vulnerable*, *elderly*, *support*, *public*, *social*,
Mitigation and Lockdown	*coronavirus*, *quarantine*, *lockdown*, *distancing*, *cancel*, *event*, *postpone*, *ban*, *curb*, *restriction*, *ministry*, *minister*, *response*, *contain*, *control*, *prevention*, *combat*, *airport*, *public*
Economic Crisis	*coronavirus*, *economy*, *economic*, *worker*, *market*, *fear*, *concern*, *affect*, *effect*, *global*, *emergency*, *industry*, *tourism*, *payment*, *hard*, *unemployment*
Death	*coronavirus*, *death*, *kill*, *toll*, *die*, *dead*, *threat*, *deadly*, *severe*, *vulnerable*

Emerging from the embers of the pandemic’s peak are nascent Recovery narratives that are regionally divergent due to the contextual nature of recovery pathways. We observe that an emerging phase—Phase 4 (May 2020) ‘*Recovery’—*in Oceania focused on ‘hope’ and ‘uncertainty’ and North America focused on ‘opening up the economy’ and ‘tackling discrimination.’

### Global convergence and regional divergence in Covid-19 narratives across six continents

By further analyzing each region’s dominant narratives, we found interesting patterns during the pandemic across 8 months. The Pre-Pandemic phase evidenced divergent disease-related topics, reflecting the unique public health concerns across different regions. The transition to the Early Pandemic and Peak Pandemic phases saw a convergence, reflecting the global and sustained influence of Covid-19 on news media narratives.

**Fig 3:**
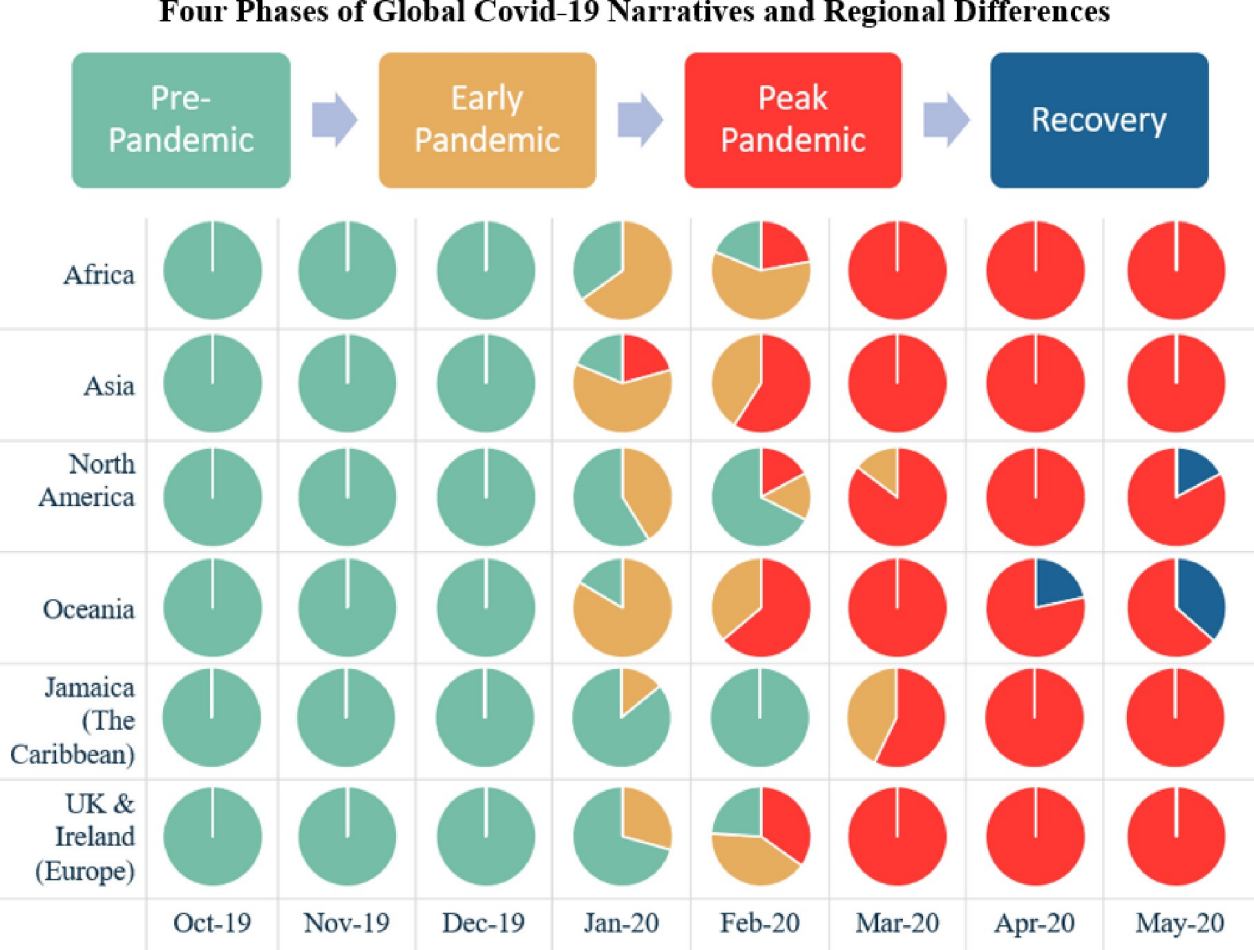
Global convergence and regional divergence in Covid-19 narratives across six continents. Pre-pandemic narratives (Oct’19–Dec’19; coded ‘green’) were divergent across regions with Africa focused on infectious diseases (e.g., monkeypox), Asia on dengue fever, Oceania on plant-related fungal diseases and North America on Lyme disease and AIDS. Early and Peak Pandemic evidenced a global convergence, reflecting the omnipresence of Covid-19 globally. The brief transition from early (coded ‘orange’) to peak (coded ‘red’) pandemic narratives underscored the pandemic’s rapid spread across six continents. Emerging from the embers of the pandemic’s peak are nascent recovery narratives (coded ‘blue’) that are regionally divergent—Oceania focused on hope and an uncertain future while North America focused on re-opening the economy and tackling discrimination.

**Fig 4:**
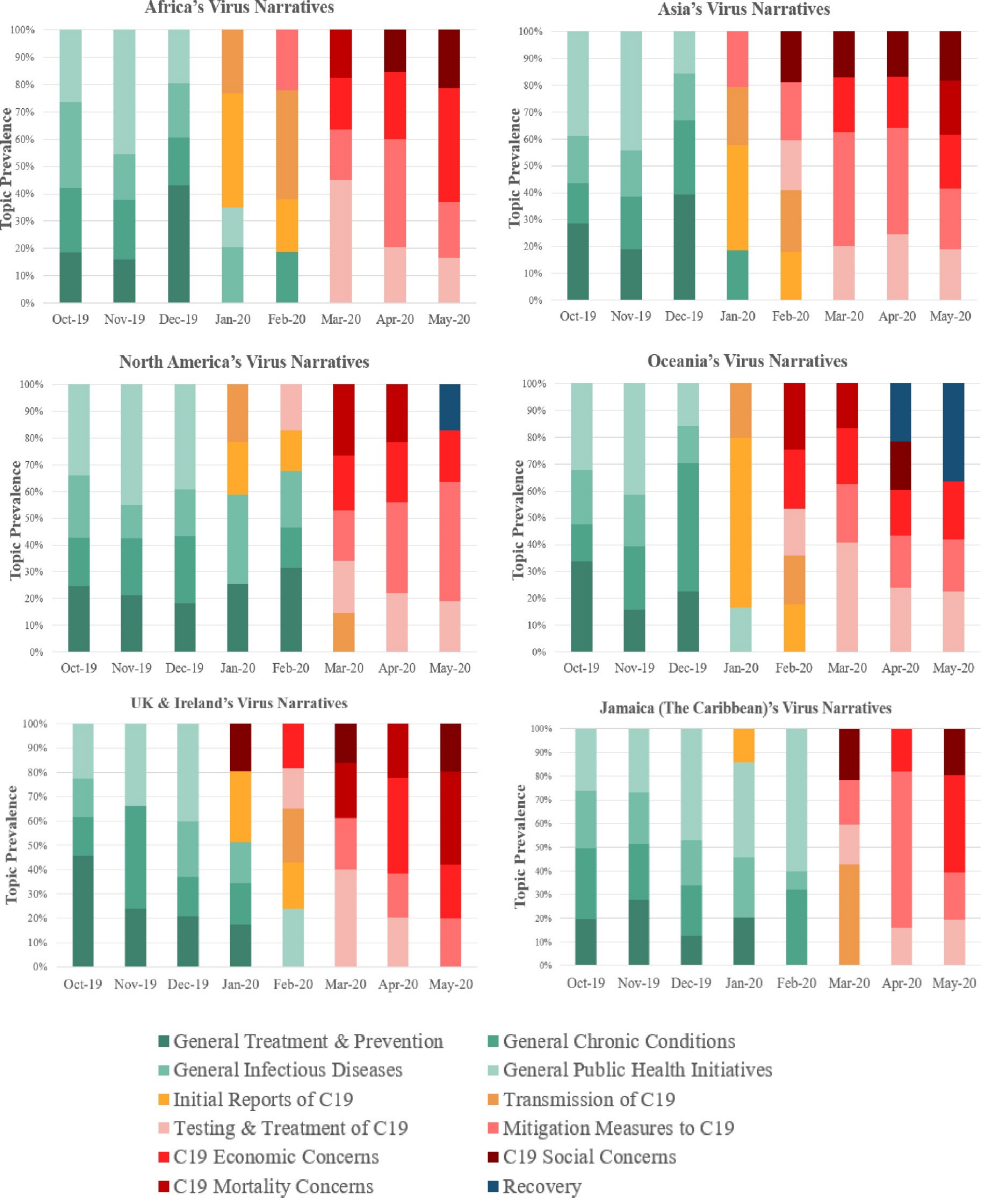
Content of regional Covid-19 narratives across six continents.

We elaborate on this ‘divergent—convergent—divergent’ patterns across 8 months from October’19 to May’20. Figs 3 and 4 present the regional differences. While all six regions exhibited the variety of subtopics at the Pre-Pandemic Phase, the regions differed in the specific *types* of illnesses these topics were focused on. For instance, in Asia, the top topic was Dengue Risk as indicated by terms like *aegypti*, *mosquito*, and *dengue*—reflecting the high incidence of dengue spread by the *Aedes Aegypti* mosquito [27]. In contrast for Oceania, Fungal Diseases dominated the narratives, articulated by *bee*, *pest*, *kauri*, *dieback*—reflecting rising incidence of fungal diseases in Australian beehives [28], and national attention on the kauri tree, a beloved indigenous tree, prone to the kauri dieback disease [29]. In North America, the joint top topics were Lyme Disease and AIDS; In UK and Ireland, it was COPD; In Africa, it was Infections Diseases like *monkey pox*, *cholera*, *and* polio; In Jamaica (the Caribbean), the top topics were Malaria and plant-related disease such as Banana Disease [30] and Citrus Greening Disease [31].

With the onset of the pandemic, however, the topical diversity across regions evaporated, and converged singularly on Covid-19 topics, especially in March 2020 (Fig 3). Beyond peak pandemic, recovery narratives begin to emerge with hints of divergence. For instance, in Oceania, such narratives made up 21.6% of societal narratives in April 2020, and 36.5% in May 2020 with hope and future uncertainty as key themes; in North America, this topic, centered around the economic re-opening and tackling discrimination, was responsible for 17% in May 2020.

## Discussion

This study provided a framework to systematically analyze the pandemic’s impact on online news media’s narratives of health and disease across 20 countries. While epidemiologic models track the progression of Covid-19 in black-and-white statistics of incidence and mortality, our framework captured the richness of Covid-19’s news media portrayals and the nuanced stories behind the numbers.

Our framework found a divergent-convergent-divergent pattern of global disease narratives across 20 countries. Before the pandemic, the divergence of news media narratives on diseases is unsurprising given the region-specific issues. From Jan to April 2020, all disease-related topics converged on Covid-19, underscoring the pandemic’s severity and global reach. In May 2020, recovery narratives emerged, albeit in a divergent manner, across different regions. For example, New Zealand and Australian’s recovery narratives focused on hope and uncertainty while the United States focused on economic re-opening and tackling discrimination—underscoring the potential difference in social impact of Covid-19 across regions.

Our study has both conceptual and practical significance. Conceptually, the few studies to dynamically track news media narratives across 8 months and 20 countries—providing an unprecedented picture of how the pandemic has shaped news content and dominated the news cycle. Most significantly, the extreme foregrounding of Covid-19 stories above other illnesses is a worrying finding, particularly with the agenda setting theory and its impact on social perception in mind. While heightened alert during a pandemic is certainly justified to prevent deaths in the population, intensified news media focus on this virus may overshadow other equally important aspects of health. For instance, studies from around the globe have reported a concerning trend in care delays during this pandemic: a dramatic decline in preventative screening for chronic disease like diabetes [32], cancer diagnosis and treatment [33], routine measures like childhood vaccinations and lead screenings [34], and treatment for tuberculosis [35]. While this situation is multi-faceted and influenced by lockdowns and the halting of non-crucial primary care services, the long-term impact of prolonged media coverage on Covid-19 may still heighten fear of this virus over seeking testing and treatment for other preventable illnesses. Instead, government intervention may suggest that news outlets pivot some focus to other virus-related issues to alleviate this worrying trend.

Further, existing survey studies [36–41] measured static attitudes [38] within specific groups and countries, that cannot capture Covid-19’s fast-moving impact across countries. Practically, our study lays the critical groundwork for scholars and policymakers to evaluate the impact of policy responses through four different phases of the pandemic.

While our study circumvented the shortcomings of survey studies, it is not without limitations. Our database consisted of only English online sources [42–44], leaving out Asian countries like China, Japan, and South Korea where many early cases were reported. This is a significant limitation that will be addressed in future studies when we expand the database to other languages. Another shortcoming is the lack of data from social media sources. The diversity of social media usage across multiple platforms makes data collation challenging, and most social media platforms such as Facebook are closed for public access—they have also become increasingly monetized, selling selected datasets that may not be representative. Nevertheless, this is significant drawback that we will overcome in future studies when we augment our database. Future iterations of the study may also consider charting how the specific keyword *Wuhan Virus* has been used in news media narratives, as it began as neutral shorthand for early coverage of this pandemic, but following the official coining of *Covid-19* on 11 February 2020 by the WHO, has been used in anti-Asian disinformation narratives.

In conclusion, our study provided a ‘front row seat’—with large-scale and dynamic data—on how news media narratives shifted with a global pandemic. Narratives matter because they shape the core tentpoles of our society that include the economy, elections, and employment, and foregrounds what a population places their focus on. We provided a data-driven four-phase framework on how society made sense of Covid-19 through the lenses of online news media—laying the important groundwork to systematically evaluate policy responses, and design better risk communication strategies.

## References

[1] Kang L, Ma S, Chen M, Yang J, Wang Y, Li R, et al. Impact on mental health and perceptions of psychological care among medical and nursing staff in Wuhan during the 2019 novel coronavirus disease outbreak: A cross-sectional study. Brain Behav Immun. 2020;87: 11–17. doi: 10.1016/j.bbi.2020.03.028 32240764 PMC7118532

[2] Casanova M, Pagani Bagliacca E, Silva M, Patriarca C, Veneroni L, Clerici CA, et al. How young patients with cancer perceive the COVID-19 (coronavirus) epidemic in Milan, Italy: Is there room for other fears? Pediatr Blood Cancer. 2020;67: e28318. doi: 10.1002/pbc.28318 32240567

[3] Taghrir MH, Borazjani R, Shiraly R. COVID-19 and Iranian Medical Students; A Survey on Their Related-Knowledge, Preventive Behaviors and Risk Perception. Arch Iran Med. 2020;23: 249–254. doi: 10.34172/aim.2020.06 32271598

[4] Khader Y, Al Nsour M, Al-Batayneh OB, Saadeh R, Bashier H, Alfaqih M, et al. Dentists’ Awareness, Perception, and Attitude Regarding COVID-19 and Infection Control: Cross-Sectional Study Among Jordanian Dentists. JMIR Public Health Surveill. 2020;6: e18798. doi: 10.2196/18798 32250959 PMC7147327

[5] Abdelhafiz AS, Mohammed Z, Ibrahim ME, Ziady HH, Alorabi M, Ayyad M, et al. Knowledge, Perceptions, and Attitude of Egyptians Towards the Novel Coronavirus Disease (COVID-19). Journal of Community Health. 2020;45: 881–890. doi: 10.1007/s10900-020-00827-7 32318986 PMC7173684

[6] McFadden SM, Malik AA, Aguolu OG, Willebrand KS, Omer SB. Perceptions of the adult US population regarding the novel coronavirus outbreak. PLOS ONE. 2020;15: e0231808. doi: 10.1371/journal.pone.0231808 32302370 PMC7164638

[7] Lohiniva A-L, Sane J, Sibenberg K, Puumalainen T, Salminen M. Understanding coronavirus disease (COVID-19) risk perceptions among the public to enhance risk communication efforts: a practical approach for outbreaks, Finland, February 2020. Eurosurveillance. 2020;25: 2000317. doi: 10.2807/1560-7917.ES.2020.25.13.2000317 32265008 PMC7140598

[8] Motta Zanin G, Gentile E, Parisi A, Spasiano D. A Preliminary Evaluation of the Public Risk Perception Related to the COVID-19 Health Emergency in Italy. International Journal of Environmental Research and Public Health. 2020;17: 3024. doi: 10.3390/ijerph17093024 32349253 PMC7246845

[9] Agarwal S, Kaushik J. Student’s Perception of Online Learning during COVID Pandemic. The Indian Journal of Pediatrics. 2020;87. doi: 10.1007/s12098-020-03327-7 32385779 PMC7205599

[10] Ng R, Chow TYJ, Yang W. Culture Linked to Increasing Ageism during Covid-19: Evidence from a 10-billion-word Corpus across 20 Countries. The Journals of Gerontology: Series B. 2021 [cited 20 Apr 2021]. doi: 10.1093/geronb/gbab057 33786581 PMC8083600

[11] Ng R, Chow TYJ. Aging Narratives over 210 years (1810–2019). The Journals of Gerontology: Series B. 2020. doi: 10.1093/geronb/gbaa222 33300996 PMC7798532

[12] Ng R, Lim WJ. Ageism linked to culture, not demographics: Evidence from an 8-billion-word corpus across 20 countries. J Gerontol B Psychol Sci Soc Sci. doi: 10.1093/geronb/gbaa181 33099600 PMC8557828

[13] Wozniak A, Willey J, Benz J, Hart N. COVID Impact Survey. Chicago, IL: National Opinion Research Center. 2020. Available from: https://www.covid-impact.org/

[14] McCombs ME, Shaw DL, Weaver DH. New Directions in Agenda-Setting Theory and Research. null. 2014;17: 781–802. doi: 10.1080/15205436.2014.964871

[15] Gerbner G, Gross L, Morgan M, Signorielli N. Growing up with television: cultivation processes. In: Jennings B, Zillmann D, editors. Media effects: advances in theory and research. New Jersey: Lawrence Erlbaum Associates; 2002. pp. 43–68. doi: 10.1080/08897070209511479

[16] Alitavoli R, Kaveh E. The U.S. Media’s Effect on Public’s Crime Expectations: A Cycle of Cultivation and Agenda-Setting Theory. Societies. 2018;8. doi: 10.3390/soc8040092 33520292 PMC7797619

[17] Iyengar Shanto, and Kinder Donald. News That Matters: Television and American Opinion. [in English] American Politics and Political Economy. Chicago: University of Chicago Press, 1987.

[18] Smith J, McCarthy JD, McPhail C, Augustyn B. From Protest to Agenda Building: Description Bias in Media Coverage of Protest Events in Washington, D.C. Social Forces. 2001;79: 1397–1423.

[19] Soroka SN. Media, Public Opinion, and Foreign Policy. Harvard International Journal of Press/Politics. 2003;8: 27–48. doi: 10.1177/1081180X02238783

[20] Smales LA. News sentiment and the investor fear gauge. Finance Research Letters. 2014;11: 122–130. doi: 10.1016/j.frl.2013.07.003

[21] Davies M. The new 4.3 billion word NOW corpus, with 4–5 million words of data added every day. The 9th International Corpus Linguistics Conference. 2017. Available from http://www.birmingham.ac.uk/Documents/college-artslaw/corpus/conferencearchives/2017/general/paper250.pdf

[22] Full-text data from English-Corpora.org: billions of words of downloadable data. [cited 5 Jun 2021]. Available: https://www.corpusdata.org/now-sources.asp

[23] Ng R, Allore HG, Trentalange M, Monin JK, Levy BR. Increasing Negativity of Age Stereotypes across 200 Years: Evidence from a Database of 400 Million Words. PLOS ONE. 2015;10: e0117086. doi: 10.1371/journal.pone.0117086 25675438 PMC4326131

[24] Blei DM, Ng AY, Jordan MI. Latent dirichlet allocation. J Mach Learn Res. 2003;3: 993–1022. doi: 10.5555/944919.944937

[25] Chanen A, Patrick J. Measuring Correlation Between Linguist’s Judgments and Latent Dirichlet Allocation Topics. Proceedings of the Australasian Language Technology Workshop 2007. Melbourne, Australia; 2007. pp. 13–20. Available from https://www.aclweb.org/anthology/U07-1005

[26] Zhang Y, Eick CF. Tracking Events in Twitter by Combining an LDA-Based Approach and a Density–Contour Clustering Approach. Int J Semant Comput. 2019;13(1): 87–110. doi: 10.1142/S1793351X1940005187

[27] World Health Organization. Dengue and severe dengue. 2020 Jun 23 [cited 25 Feb 2021]. Available from https://www.who.int/news-room/fact-sheets/detail/dengue-and-severe-dengue

[28] Nobel E, Ridnell J. Chalkbrood fungal disease on the rise in Australian beehives. Abc News. 2019 March 28 [cited 25 Feb 2021]. Available from https://www.abc.net.au/news/2019-03-28/chalkbrood-fungal-disease-on-the-rise-in-australian-beehives/10945502

[29] Keep Kauri Standing. What is kauri dieback disease? 2016 [cited 25 Feb 2021]. Available from https://www.kauridieback.co.nz/what-is-kauri-dieback/

[30] Balford H. Jamaica on alert for dreaded banana disease. Jamaica Observer. 2019 Oct 9 [cited 25 Feb 2021]. Available from http://www.jamaicaobserver.com/article/20191009/ARTICLE/191009691/1470

[31] Smith-Edwards A. Government continues measures to tackle citrus greening disease. Jamaica Information Service. 2014 Nov 13 [cited 25 Feb 2021]. Available from https://jis.gov.jm/government-continues-measures-tackle-citrus-greening-disease/

[32] Wright A, Salazar A, Mirica M, Volk LA, Schiff GD. The Invisible Epidemic: Neglected Chronic Disease Management During COVID-19. J GEN INTERN MED. 2020;35: 2816–2817. doi: 10.1007/s11606-020-06025-4 32666485 PMC7359916

[33] Cancer Diagnosis and Treatment Upended by COVID-19, Says CRUK. In: Medscape [Internet]. [cited 5 Jun 2021]. Available: http://www.medscape.com/viewarticle/929575

[34] ‘Menu of neglect’: the long-term health problems being ignored in US amid pandemic. In: the Guardian [Internet]. 26 Apr 2021 [cited 5 Jun 2021]. Available: http://www.theguardian.com/us-news/2021/apr/26/us-covid-coronavirus-health-resources-preventative-care

[35] India: Excessive focus on COVID-19 ignores other diseases. [cited 5 Jun 2021]. Available: https://www.aa.com.tr/en/asia-pacific/india-excessive-focus-on-covid-19-ignores-other-diseases/2061296

[36] Heidekrueger PI, Juran S, Szpalski C, Larcher L, Ng R, Broer PN. The current preferred female lip ratio. Journal of Cranio-Maxillofacial Surgery. 2017;45: 655–660. doi: 10.1016/j.jcms.2017.01.038 28318919

[37] Sima LC, Ng R, Elimelech M. Modeling Risk Categories to Predict the Longitudinal Prevalence of Childhood Diarrhea in Indonesia. The American Journal of Tropical Medicine and Hygiene. 2013;89: 884–891. doi: 10.4269/ajtmh.12-0540 24019442 PMC3820331

[38] Ng R, Rayner S. Integrating psychometric and cultural theory approaches to formulate an alternative measure of risk perception. Innovation: The European Journal of Social Science Research. 2010;23: 85–100. doi: 10.1080/13511610.2010.512439

[39] Ng R, Levy B. Pettiness: Conceptualization, measurement and cross-cultural differences. PLOS ONE. 2018;13: e0191252. doi: 10.1371/journal.pone.0191252 29385157 PMC5791981

[40] Ng R, Allore HG, Monin JK, Levy BR. Retirement as Meaningful: Positive Retirement Stereotypes Associated with Longevity. Journal of Social Issues. 2016;72: 69–85. doi: 10.1111/josi.12156 27346893 PMC4920366

[41] Ng R, Allore HG, Levy BR. Self-Acceptance and Interdependence Promote Longevity: Evidence From a 20-year Prospective Cohort Study. International Journal of Environmental Research and Public Health. 2020;17: 5980. doi: 10.3390/ijerph17165980 32824658 PMC7460297

[42] Ng R, Indran N. Role-Based Framing of Older Adults Linked to Decreased Ageism Over 210 Years: Evidence From a 600-Million-Word Historical Corpus. The Gerontologist. 2021 [cited 9 Aug 2021]. doi: 10.1093/geront/gnab108 34323967 PMC9019650

[43] Ng R, Indran N. Societal perceptions of caregivers linked to culture across 20 countries: Evidence from a 10-billion-word database. PLOS ONE. 2021;16: e0251161. doi: 10.1371/journal.pone.0251161 34197470 PMC8248619

[44] Ng R. Cloud Computing in Singapore: Key Drivers and Recommendations for a Smart Nation. Politics and Governance. 2018;6: 39–47. doi: 10.17645/pag.v6i4.1757

